# Surgical Wards—Service of Dr. E. Peace

**Published:** 1841-11-13

**Authors:** 


### CLINICAL REPORTS.

### PENNYSLVANIA HOSPITAL.

*Surgical Wards—Service of Dr. E. Peace.*

*Cases discharged*, *Oct.* 27.—1. G. W., æt. 31, admitted July 14, 1841, for deep syphilitic ulceration of the soft palate and fauces of five or six weeks duration. Treated until September 13th internally with syr. sarsap. comp., and locally with the frequent application of the solid nitrate of silver, and the constant use of astringent gargles, without much material or permanent benefit. The syr. sarsap. comp., and all local applications, but a simple gargle of slippery elm mucilage, were then omitted, and the following prescription ordered:

℞. Potass. Iodid. ℈jss.
Aquæ, ℥ss.
Bis. die.

This allowance was gradually increased in the course of three weeks to ℈jss. four times a day, and so continued, without giving rise to any unpleasant effects for about two weeks longer; when the man, whose improvement under the remedy had been immediate and rapid, was discharged, to all appearance cured.

Hydriodate of potash in large doses, is a favourite and very efficient prescription of Ricord in Paris for all cases of tertiary syphilis. It was tried, with equally striking good effects, upon another case of analogous but more obstinate character in the same wards of this hospital.

A third patient, in whom there was no ulceration of any account, and for whom the remedy was prescribed in doses of gr. xv. twice a day, derived no advantage from it.

2.—S. W., negress, æt. 21, admitted October 12 for lacerated wound of face, produced by a glass tumbler thrown with sufficient force to break upon the part. She presented a very open ragged cut, at least four inches long, beginning on the forehead above the left eyebrow, curving down across the root of the nose near the inner canthus of the left eye, and terminating on the cheek of that side. The pendulous margins of the wound were brought together with four sutures and some adhesive strips. The hæmorrhage was easily controlled by compression. The wound suppurated freely, and was dressed with cataplasms. Two spicula of glass were removed at the third dressing; the sutures all sloughed away on the third day; the ragged edges of the laceration were rounded off on the fourth; and by the twelfth, cicatrization was complete, except in a portion near the internal canthus of about the circumference of a ten cent piece. This part was touched with nitrate of silver, and dressed with Turner's cerate. Cure effected in 15 days.

3.—M. A. P., negress, æt. 21, intemperate; admitted October 18. A case of lacerated wound, about fifteen lines long, and six deep, immediately over the right eyebrow, and the result of a blow with a stick of wood. This was attended with great inflammation of the adjacent parts, and subsequent free suppuration. Poulticed six days, and then dressed with adhesive strips, and a compressing bandage two days, until she was discharged at her own request. This patient was threatened with delirium tremens, on which account she was allowed, during the first week, a small portion of stimulus, which was gradually reduced.

4.—L. J., negro, æt. 14, admitted October 6, for lacerated right hand from the bursting of a pistol. Palm of the hand, the fore and middle finger and thumb, very much shattered; the latter torn off, except by a string of integuments; all the metacarpal bones fractured and exposed; the joint opened, and the large branches of the radial artery ruptured. Amputation by the circular operation was performed just above the joint within three hours after the accident. The boy fell asleep directly the stump was dressed; the dressings were not renewed until the fourth day, and the wound healed by the first intention, without a single uncomfortable symptom. There were but two ligatures; one came away on the 13th, and the other on the 14th day, the holes of exit being so small as only to allow them to be withdrawn with difficulty. Cured in twenty days.

5.—*Oct. 28th.* S. G., æt. 26, butcher, temperate, admitted September 26th with extensive laceration of the left hand, resulting from the bursting of a gun. The thumb was nearly detached and much shattered, the palm of the hand greatly torn up, the first and second metacarpal bones broken and laid bare, the wrist joint opened, the radial artery exposed, and the ulnar artery ruptured. Amputation above the wrist by the circular method was performed within four hours and a half after the accident. Three ligatures were used and a light dressing applied. The stump was re-dressed the second day after the operation, and every day thereafter except the fourth, when it was omitted on account of a disposition to hæmorrhage at the previous dressing. The wound granulated healthily, with very moderate suppuration. The first ligature was removed on the 13th day, the second on the 17th day, and the third on the 19th day. Cured in 32 days. E. H.

